# Repurposing Selamectin as an Antimicrobial Drug against Hospital-Acquired *Staphylococcus aureus* Infections

**DOI:** 10.3390/microorganisms11092242

**Published:** 2023-09-06

**Authors:** Veronica Folliero, Federica Dell’Annunziata, Biagio Santella, Emanuela Roscetto, Carla Zannella, Nicoletta Capuano, Alessandro Perrella, Anna De Filippis, Giovanni Boccia, Maria Rosaria Catania, Massimiliano Galdiero, Gianluigi Franci

**Affiliations:** 1 Department of Medicine Surgery and Dentistry, University of Salerno, 84081 Baronissi, Italy; vfolliero@unisa.it (V.F.); federica.dellannunziata@unicampania.it (F.D.); bi.santella@gmail.com (B.S.); capuanonicoletta95@gmail.com (N.C.); gboccia@unisa.it (G.B.); 2 Department of Experimental Medicine, University of Campania “Luigi Vanvitelli”, 80138 Naples, Italy; carla.zannella@unicampania.it (C.Z.); anna.defilippis@unicampania.it (A.D.F.); massimiliano.galdiero@unicampania.it (M.G.); 3 Department of Molecular Medicine and Medical Biotechnology, University of Naples Federico II, 80138 Naples, Italy; emanuela.roscetto@unina.it (E.R.); mariarosaria.catania@unina.it (M.R.C.); 4 Division Emerging Infectious Disease and High Contagiousness, Hospital D Cotugno, 80131 Naples, Italy; alessandro.perrella@ospedalideicolli.it; 5 Clinical Pathology and Microbiology Unit, San Giovanni di Dio e Ruggi D’Aragona University Hospital, 84126 Salerno, Italy; 6 Hospital Hygiene and Epidemiology Complex Operating Unit, San Giovanni di Dio e Ruggi D’Aragona University Hospital, 84126 Salerno, Italy; 7 Section of Microbiology and Virology, University Hospital “Luigi Vanvitelli”, 80138 Naples, Italy

**Keywords:** *Staphylococcus aureus*, antimicrobial resistance, drug repurposing, anthelmintic drugs, macrolides, biofilm

## Abstract

The emergence of multidrug-resistant strains requires the urgent discovery of new antibacterial drugs. In this context, an antibacterial screening of a subset of anthelmintic avermectins against gram-positive and gram-negative strains was performed. Selamectin completely inhibited bacterial growth at 6.3 μg/mL concentrations against reference gram-positive strains, while no antibacterial activity was found against gram-negative strains up to the highest concentration tested of 50 μg/mL. Given its relevance as a community and hospital pathogen, further studies have been performed on selamectin activity against *Staphylococcus aureus* (*S. aureus*), using clinical isolates with different antibiotic resistance profiles and a reference biofilm-producing strain. Antibacterial studies have been extensive on clinical *S. aureus* isolates with different antibiotic resistance profiles. Mean MIC_90_ values of 6.2 μg/mL were reported for all tested *S. aureus* strains, except for the macrolide-resistant isolate with constitutive macrolide-lincosamide-streptogramin B resistance phenotype (MIC_90_ 9.9 μg/mL). Scanning Electron Microscopy (SEM) showed that selamectin exposure caused relevant cell surface alterations. A synergistic effect was observed between ampicillin and selamectin, dictated by an FIC value of 0.5 against methicillin-resistant strain. Drug administration at MIC concentration reduced the intracellular bacterial load by 81.3%. The effect on preformed biofilm was investigated via crystal violet and confocal laser scanning microscopy. Selamectin reduced the biofilm biomass in a dose-dependent manner with minimal biofilm eradication concentrations inducing a 50% eradication (MBEC_50_) at 5.89 μg/mL. The cytotoxic tests indicated that selamectin exhibited no relevant hemolytic and cytotoxic activity at active concentrations. These data suggest that selamectin may represent a timely and promising macrocyclic lactone for the treatment of *S. aureus* infections.

## 1. Introduction

**Figure 1:**
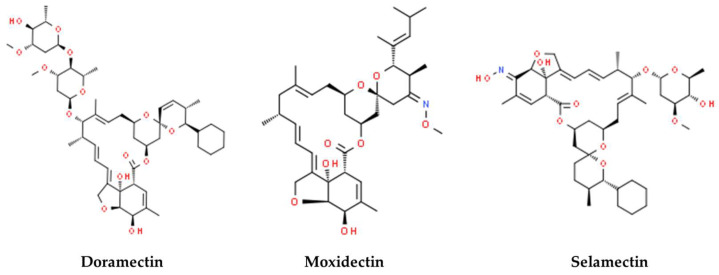
Avermectin drugs used in this study. Images were obtained from ChemSpider.

The emergence of hospital-acquired infections (HAIs) represents a serious and continual threat to public health in both developed and developing nations [1]. These infections have garnered a pervasive global presence, yielding direct and indirect socio-economic repercussions [2]. According to the Centers for Disease Control and Prevention (CDC), a distressing 10 out of every 100 patients fall victim to HAIs, leading to fatal outcomes in a striking 87.1% of cases [3]. Furthermore, recent estimates by the CDC indicate annual healthcare costs ranging from 28 to 45 billion dollars in the United States. The World Health Organization (WHO) has underscored the presence of 12 bacterial families, grouped into three distinct categories based on the exigency for novel antibiotics (critical, high, and medium priority). Among those deemed high priority is methicillin-resistant *Staphylococcus aureus* (MRSA) [4]. This bacterial strain stands as the primary etiology of the lower respiratory tract and surgical site infections, emerging as the second leading source of bacteremia, cardiovascular infections, and infections stemming from indwelling medical devices, both within the community and notably within the hospital environment [5]. Although current antibiotic regimens, including vancomycin, daptomycin, and linezolid, target MRSA, resistance to these drugs is swiftly emerging [6]. Beyond its antibiotic resistance, addressing MRSA infections is further compounded by its propensity to establish biofilms on both biotic and abiotic surfaces, alongside its ability to subsist within host cells [7]. These growth modalities can substantially undermine the efficacy of host defenses and antibiotic therapies [8]. Annually, nearly 25,000 patients within Europe continue to succumb to severe infections caused by antibiotic-resistant bacteria, with a notable proportion attributed to MRSA. Consequently, the imperative to search for innovative therapeutic antimicrobial strategies capable of countering both antibiotic-resistant and persistent MRSA strains is underscored. Current paradigms of antimicrobial drug discovery encompass various stages, including microbiological in vitro antibacterial assays, structure-activity relationship studies, and eventual in vivo experimental assessments to ascertain toxicity and performance [9]. However, this protracted and financially demanding process (with costs reaching up to ~$800 million and spanning 15–20 years) coupled with the swift emergence of antimicrobial resistance has led to waning interest in the pursuit of new antibiotics [3,10]. In response, renewed attention has focused on repurposing pre-existing drugs that have previously received approval from the Food and Drug Administration (FDA) as antibiotics. Indeed, drug repurposing offers a novel and potent avenue for drug development, capitalizing on extant knowledge of their pharmacology, toxicity, and formulation [11]. Drug repurposing affords several advantages over conventional methods, encompassing marked reductions in research and development costs, expedited FDA approval timelines, and potential market exclusivity [12]. Numerous evidence has documented the antibacterial efficacy of various anthelmintic drugs against a spectrum of pathogens, including strains of Staphylococcus and Enterococcus species, *Acinetobacter baumannii*, *Pseudomonas aeruginosa* (*P. aeruginosa*), *Escherichia coli* (*E. coli*), *Clostridium difficile* and others [13]. Anthelmintics constitute a category of anti-parasitic agents primarily designated for worm infections [14]. Within this category, the avermectins assume prominence, featuring 16 macrocyclic lactone derivatives produced through *Streptomyces avermitilis* fermentation [15]. While structurally akin to antibacterial macrolides and antifungal macrocyclic polygenes, the avermectins diverge in terms of their mechanism of action. These derivatives are classified into four major (A1a, A2a, B1a, and B2a) and minor (A1b, A2b, B1b, and B2b) classes. Notable members encompass ivermectin, abamectin, nemadectin, doramectin, milbemycin, eprinomectin, moxidectin, and selamectin. The molecular structures of select avermectins are depicted in Figure 1. Limited evidence exists concerning the antibacterial potential of doramectin, moxidectin, and selamectin [16]. These molecules interact with gamma-aminobutyric (GABA) receptors and glutamate-dependent chloride channels within the nervous systems of parasites. As these channels are indispensable for parasite survival, their disruption leads to compromised neurotransmission, culminating in paralysis and invertebrate demise [17]. The rise of multidrug-resistant pathogens poses a pressing threat to public health, challenging healthcare workers with a dwindling arsenal of effective antibiotics. This has led to the emergence of complex infections that prove difficult to handle. Infections caused by *Staphylococcus aureus* (*S. aureus)* have shown particularly serious implications. In response to this growing crisis, there is a compelling need to expedite the advancement of novel antibiotics. However, it is crucial to recognize that this research is both financially demanding and time-consuming. In this context, the main objective of this study was to examine the antibacterial potentials of three commercially available non-antibiotic drugs against *S. aureus* infections. This effort seeks to establish an alternative avenue for addressing S. aureus infections through a rapid and cost-effective drug discovery strategy. The latter, combined with catalytic environmental corrections, would significantly limit the spread of mutant-resistant bacteria and the risk of looming in complex infections.

## 2. Materials and Methods

### 2.1. Compound

Doramectin, moxidectin, and selamectin were bought by Sigma-Aldrich (Burlington, MA, USA). The compounds were dissolved in DMSO at a concentration of 1 mg/mL.

### 2.2. Characterization of the Bacterial Strains

Reference strains of *S. aureus* (ATCC 6538), *Corynebacterium striatum* (*C. striatum*, ATCC BAA-1293), *Staphylococcus epidermidis* (*S. epidermidis*, ATCC 12228), *E. coli* (ATCC 11229), *Klebsiella pneumoniae* (*K. pneumoniae*, ATCC 10031), and *P. aeruginosa* (ATCC 9027) were used for initial screening of the selected avermectins. Reference strains were purchased from the American Type Culture Collection (Manassas, VA, USA). Further exploration of the antibacterial efficacy of selamectin encompassed clinical isolates of *S. aureus* (CI1–5) characterized by diverse antimicrobial susceptibility profiles. These clinical isolates were derived from a range of sources (blood, sputum, wound, and eye) and were part of an anonymous collection established at the Microbiology Laboratory of Luigi Vanvitelli University Hospital in Naples, Italy. For the assessment of selamectin’s anti-biofilm activity, *S. aureus* reference strain ATCC 1167 was employed (Table 1). Samples were plated on Columbia agar with 5% sheep blood and Chapman’s medium (bioMerieux, Marcy-l’Etoile, France) and incubated overnight at 37 °C (Biocompare, 311DS incubator, South San Francisc, CA, USA). The identification of bacterial strains and susceptibility testing were executed through Matrix Assisted Laser Desorption Ionization-Time of Flight MS (MALDI-TOF) MS (Microflex LRF, Bruker Daltonics, Billerica, MA, USA) and the Phoenix BD system (M50 instrument, Becton Dickinson, Franklin Lakes, NJ, USA) [18,19].

### 2.3. Bacterial Growth Conditions

Bacterial strains were grown in Mueller Hinton (MH) (non-biofilm forming *C. striatum*, *S. epidermidis*, *E. coli*, *K. pneumoniae*, *P. aeruginosa*, *S. aureus* and clinical isolates CI1–5) and Luria Bertani (LB) (biofilm forming *S. aureus* ATCC 1167) media (Oxoid, Basingstoke, Hampshire, MA, USA) at 37 °C in aerobic conditions. To obtain a bacterial suspension suitable for antibacterial assays, fresh colonies of each strain, grown on MH and LB agar, were inoculated in MH and LB media, and incubated at 37 °C overnight. The bacterial suspension was resuspended in a fresh medium and further incubated at 37 °C until bacterial growth reached the exponential phase. Serial dilutions were performed to achieve the necessary bacterial load for the tests (1 × 10^6^ CFU/mL).

### 2.4. Cell Culture Conditions

The immortalized human keratinocytes (HaCaT) were employed for cytotoxicity assays and bacterial invasion tests. They were grown in high glucose DMEM medium (Gibco Life Technologies, Paisley, Scotland, UK), supplemented with 10% Fetal Bovine Serum (Gibco Life Technologies, Scotland, UK) and 1% penicillin-streptomycin solution (Gibco Life Technologies, Scotland, UK). The cells were maintained at a temperature of 37 °C, with a CO_2_ concentration of 5% in a humid environment using a ThermoFisher Forma Series II Water-Jacketed CO_2_ Incubator (Waltham, MA, USA).

### 2.5. Antibacterial Susceptibility Assays

The antibacterial activity assays were conducted using the broth microdilution method, according to the Clinical and Laboratory Standards Institute (CLSI). Assays were conducted in 96-well plates (Becton Dickinson, Franklin Lakes, NJ, USA) for a final test volume of 100 μL. Doramectin, moxidectin, and selamectin were selected and tested on *S. aureus* ATCC 6538. For each compound, dilutions in the concentration range of 50 to 0.4 μg/mL were prepared. A 1 × 10^6^ CFU/mL bacterial inoculum was incubated with the test compounds at 37 °C for 20 h under aerobic conditions. Vancomycin and meropenem (Burlington, MA, USA) were employed as positive controls (CTR+) for gram-positive and gram-negative bacteria, respectively, while untreated bacteria were considered as the negative control (CTR-). The turbidity was measured via a microplate reader (Tecan life science, Seestrasse, Switzerland). The rate of growth inhibition was determined using the following formula [20]:

$$\%\;\mathrm{Growth}\;\mathrm{inhibition}=100-\left[\frac{\left(100\;\times \;\mathrm{Abs}\;600\;\mathrm{nm}\;\mathrm{of}\;\mathrm{the}\;\mathrm{test}\;\mathrm{sample}\right)}{\mathrm{Abs}\;600\;\mathrm{nm}\;\mathrm{of}\;\mathrm{CTR}-}\right]$$

Subsequently, the antibacterial potential of active drugs was investigated more thoroughly on *S. aureus.*

### 2.6. Cell Cytotoxicity Test

The cytotoxicity assays were performed using the MTT (3-[4,5-dimethylthiazol-2-yl]-2,5 diphenyl tetrazolium bromide) method. HaCaT cells were seeded into a 96-well flat-bottom plate at a density of 2 × 10^4^ cells per well. This plate was subsequently incubated for 20 h under conditions of 37 °C, 95% humidity, and 5% CO_2_. Cells were treated with selamectin for 20 h at concentrations ranging from 50 to 0.4 μg/mL in a final volume of 100 μL. On the other hand, only the medium and DMSO were employed as CTR- and CTR+ controls, respectively. Later, 100 µL of the MTT solution (5 mg/mL) was added to each well and the plate was incubated for 3 h. Next, the medium was removed and 100 µL of DMSO was added to dissolve the formazan crystals. The amount of formazan crystal was determined by measuring the absorbance at 570 nm using a multiplate reader. The data were presented as a percentage of cell viability relative to the positive control. The levels of viability were calculated using the subsequent formula [21]:

$$\%\;\mathrm{Cytotoxicity}=100-\left[\frac{\left(\mathrm{Abs}\;570\;\mathrm{nm}\;\mathrm{of}\;\mathrm{the}\;\mathrm{test}\;\mathrm{sample}\;\times \;100\right)}{\left(\mathrm{Abs}\;570\;\mathrm{nm}\;\mathrm{of}\;\mathrm{CTR}-\right)}\right]$$

### 2.7. Hemolysis Assays

Selamectin was tested on human erythrocytes of blood group 0 obtained from a healthy patient. The blood was centrifuged at 5000 rpm for five minutes (Microfuge 16, Beckman coulter, Brea, CA, USA) and the erythrocytes were washed 5 times with a solution (TBS) containing 50 Mm Tris-HCl (pH 7.6) and 0.15 M NaCl. After the washes, the erythrocytes were diluted 10-fold using the TBS solution. A volume of 50 µL of drug at the concentrations mentioned above (50 to 0.4 μg/mL) was added to 50 µL of cell suspension and incubated at 37 °C for 1 h. Two controls were included in the assay, which were dissolved drug solvent and 0.1% Triton X-100, used as CTR- and CTR+, respectively. After incubation, the plate was centrifuged at 500× *g* for 5 min and 50 μL of supernatant from each well was transferred to a new 96-well plate (Microfuge 16, Beckman coulter, Brea, CA, USA). The supernatants were used to measure the absorbance of the released hemoglobin at 540 nm. The hemolysis percentage of each sample was calculated using the following formula:

$$\%\;\mathrm{Hemolysis}=\left[\frac{\left(\mathrm{Abs}\;540\;\mathrm{nm}\;\mathrm{of}\;\mathrm{the}\;\mathrm{test}\;\mathrm{sample}-\;\mathrm{Abs}\;540\;\mathrm{nm}\;\mathrm{of}\;\mathrm{CTR}-\right)}{(\mathrm{Abs}\;540\;\mathrm{nm}\;\mathrm{of}\;\mathrm{CTR}+-\mathrm{Abs}\;540\;\mathrm{nm}\;\mathrm{of}\;\mathrm{CTR}-}\right]\times 100$$

### 2.8. Killing Kinetic Assays

The antibacterial potential of selamectin was further evaluated through time-killing curve analysis. Dilutions in the concentration range of 3.1–100 µg/mL were assembled for a final volume of 2 mL/tube. Untreated bacteria and vancomycin were regarded as CTR- and CTR+, respectively. A bacterial inoculum of 1 × 10^6^ CFU/mL was added to each tube and then incubated at 37 °C for 20 h. Volumes of 100 µL were extracted from the bacterial suspensions and subjected to serial dilution within MH broth. The dilutions were plated on MH agar and the plates were incubated at 37 °C overnight. The resulting colonies were counted, and the CFU/mL values were determined.

### 2.9. Scanning Electron Microscopy (SEM)

Morphological changes in response to exposure to selamectin were evaluated by SEM. Bacterial samples treated with the drug solvent or vancomycin were respectively designated as CTR- and CTR+. The bacterial suspensions were fixed with 2.5% glutaraldehyde and dehydrated in 25%, 50%, 75%, 95%, and 100% ethanol. Afterwards, the bacteria were deposited on a glass support and coated with a thin layer of Au-Pd (Sputter Coater Denton Vacuum Desk V, Moorestown, NJ, USA). Morphological properties of bacterial cells were marked using an FEI Nova NanoSEM 450 at an acceleration voltage of 5 kV with Everhart Thornley Detector (ETD) and Through Lens Detector (TLD) at 10,000, 25,000, and 35,000× magnification.

### 2.10. Checkerboard Tests

Checkerboard assays were conducted to assess the synergistic effects of selamectin (A) and ampicillin (Burlington, MA, USA) (B). Double serial dilutions of the antibiotic and selamectin were assembled for the concentration ranges of 250–0.5 μg/mL and 50–0.4 μg/mL, respectively. A 1 × 10^6^ CFU/mL bacterial inoculum of *S. aureus* CI5 was added to the alone and combination compounds and the resulting plate was incubated at 37 °C for 20 h. The combined inhibitory potential was quantified through the fractional inhibitory concentration index (FICI), calculated employing the ensuing formula:

$$\mathrm{FICI}=\frac{\mathrm{MIC}\;A\;\mathrm{in}\;\mathrm{combination}}{\mathrm{MIC}\;A\;\mathrm{alone}}+\frac{\mathrm{MIC}\;B\;\mathrm{in}\;\mathrm{combination}}{\mathrm{MIC}\;B\;\mathrm{alone}}\;$$

The FICI values determine: (i) synergy, FICI ≤ 0.5; (ii) partial synergy 0.5 < FICI ≤ 1.0; (iii) no interaction 1.0 > FICI ≤ 4.0; and (iv) antagonism FICI > 4.0. The synergistic combination was further evaluated through the time-killing curve analysis. Selamectin (3.1 µg/mL) and Ampicillin (1 µg/mL) were assembled for a final volume of 2 mL/tube. Untreated bacteria, Ampicillin at the concentration of 250 and 1 µg/mL and selamectin at 6.3 and 3.1 µg/mL consisted of CTR- and CTR+. A bacterial inoculum of 2 × 10^5^ CFU/mL was inoculated in each tube and incubated at 37 °C for 20 h. Serial dilutions of the treated and untreated samples in MH broth were performed and plated on MH agar. The colonies were counted and the CFU/mL values were obtained [22].

### 2.11. In Silico Molecular Docking

The interaction of selamectin with known macrolide target was investigated through PatchDock Beta 1.3 Version (https://bioinfo3d.cs.tau.ac.il/PatchDock/php.php (accessed on 1 December 2021)) and FireDock web server (https://bioinfo3d.cs.tau.ac.il/FireDock/php.php (accessed on 3 Dicember 2021)) software. The 23S RNA structure derived from an *S. aureus* wild-type and resistant to macrolides was used to conduct the docking studies. PatchDock uses the complementarity of the molecular surfaces of the target and the ligand (selamectin) to generate the best forms of interaction. This server provided a list of candidate complexes showing: (i) the number of identified interactions; (ii) geometric complementarity score (score); (iii) approximate interface area of the complex (area); (iv) atomic contact energy (ace). Later, FireDock optimized the solutions provided by the first server, allowing the movement of the target molecule into the binding site, and changing its orientation.

### 2.12. Gentamicin Protection Assay

The HaCat cell line was used to examine the inhibition of *S. aureus* CI5 (MRSA strains) invasion by selamectin. Cells were seeded in a 12-well flat bottom plate at a density of 1.5 × 10^5^ cells per well, followed by a 24-h incubation period. After 2 h of starvation, a 3 × 10^7^ CFU/mL bacterial inoculum of *S. aureus* CI5 in antibiotic and serum-free DMEM was added to cell monolayers for 3 h at 37 °C with 5% CO_2_. Post-infection, HaCat cells were treated with DMEM supplemented with 100 μg/mL of gentamicin (Sigma-Aldrich, Burlington, MA, USA) and incubated for 2 h to eliminate extracellular bacteria. Later, the cell monolayer underwent a double wash with 1×PBS and treatment with selamectin in the concentration range of 6.3 to 0.4 μg/mL for 0.5, 1, and 2 h at 37 °C with 5% CO_2_. CTR+ and CTR- consisted of infected and uninfected cells with the bacterial inoculum, respectively. After the treatment, the cells were lysed with cold 0.1% TritonX (Sigma-Aldrich, Burlington, MA, USA) for 5 min. Serial dilutions of free bacteria were plated on MH agar and incubated overnight at 37 °C. The ensuing colonies were enumerated to calculate CFU/mL values. The outcomes were expressed as a percentage of the intracellular bacterial load.

### 2.13. Biofilm Degradation Assay

The potential of selamectin to degrade preformed biofilms was evaluated through the crystal violet (CV) test. A bacterial inoculum of 2 × 10^8^ CFU/mL in LB supplemented with 1% glucose (Sigma-Aldrich, Burlington, MA, USA) was prepared. After, 100 µL of the bacterial suspension was added to each well of a 96-well plate and incubated at 37 °C for a period of 24 h under static conditions, promoting biofilm growth. After incubation, planktonic cells were removed by washing with 1×PBS and the mature biofilm was treated with selamectin at concentrations from 0.39 to 50 μg/mL. Biofilms grown without selamectin were used as CTR- and CTR+ was represented by biofilm treated with vancomycin at 512 μg/mL. After 24 h of treatment, the biofilm was washed with 1×PBS, and the biomass of the biofilm was quantified by adding 100 µL of 0.1% CV (Sigma-Aldrich, Burlington, MA, USA) to each well for 30 min at room temperature with agitation. The dye was removed by washing with 1×PBS. The solubilization of the biofilm took place with 98% ethanol for 40 min at room temperature under agitation. The absorbance at 570 nm was obtained via a microplate reader. The minimum biofilm eradication concentration (MBEC) was calculated according to the following formula:

$$\%\;\mathrm{Biofilm}\;\mathrm{degradation}=100-\left[\frac{\left(\mathrm{Abs}\;570\;\mathrm{nm}\;\mathrm{of}\;\mathrm{the}\;\mathrm{test}\;\mathrm{sample}\right)}{\mathrm{Abs}\;570\;\mathrm{nm}\;\mathrm{of}\;\mathrm{CTR}-}\right]\times 100$$

### 2.14. Confocal Laser Scanning Microscopy Analysis

Confocal laser scanning microscopy was exploited to evaluate the effects of selamectin on the preformed biofilm of the reference biofilm-producing *S. aureus* strain. Biofilms were grown on Nunc^®^ Lab-Tek^®^ II chamber slides (Sigma-Aldrich, Burlington, MA, USA) and treated with concentrations of 6.25 µg/mL or solvent control. Control biofilms were grown without selamectin. Biofilm cells were stained with the LIVE/DEAD BacLight Bacterial Viability Kit (Molecular Probes, Eugene, OR, USA), containing SYTO 9 dyes and propidium iodide (PI). Image acquisition was executed utilizing an inverted confocal laser scanning microscope (LSM 710, Carl Zeiss, Oberkochen, Germany), with subsequent analysis performed employing Z-Stack software Z-STACK 3.0.2. In this process, a series of optical sections with a thickness of 1 µm each were sequentially captured along the *z*-axis across the entire biofilm specimen.

### 2.15. Statistic Analysis

The tests were performed in biological triplicate and expressed as mean ± standard deviation (SD). 50% minimum inhibitory concentration (MIC_50_), 90% minimum inhibitory concentration (MIC_90_), 50% cytotoxic concentration (CC_50_), and 50% minimum biofilm eradication concentration (MBEC_50_) values were calculated from the dose-effect curves by non-linear regression analysis via the software Graph Pad Prism 9.0 (San Diego, CA, USA). The significance of the difference between treated samples and CTR- was obtained via one-way analysis of variance (ANOVA) with Dunnett’s test as post hoc by the software Graph Pad Prism 9.0 (USA). A *p*-value < 0.05 was considered significant.

## 3. Results

### 3.1. Antibacterial Activity

**Figure 2:**
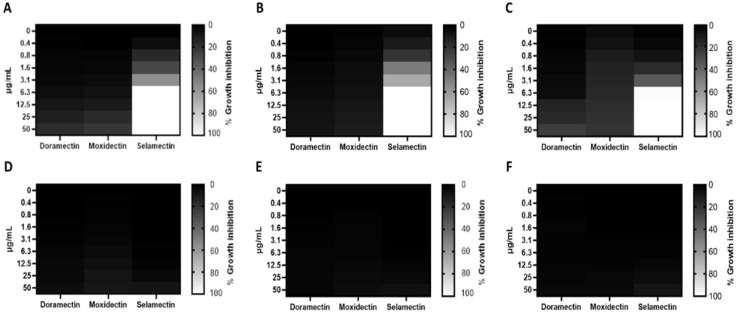
Antibacterial potential of avermectin drugs. Heatmap representations of bacterial inhibition after treatment of *S. aureus* (**A**), *C. striatum* (**B**), *S. epidermidis* (**C**), *E. coli* (**D**), *K. pneumoniae* (**E**), and *P. aeruginosa* (**F**) with doramectin, moxidectin, selamectin (50–0.4 µg/mL) for 20 h. Vancomycin and meropenem were used as CTR+ for Gram-positive and -negative bacteria, while untreated bacteria were considered the CTR-. The *p*-value was <0.05.

**Figure 3:**
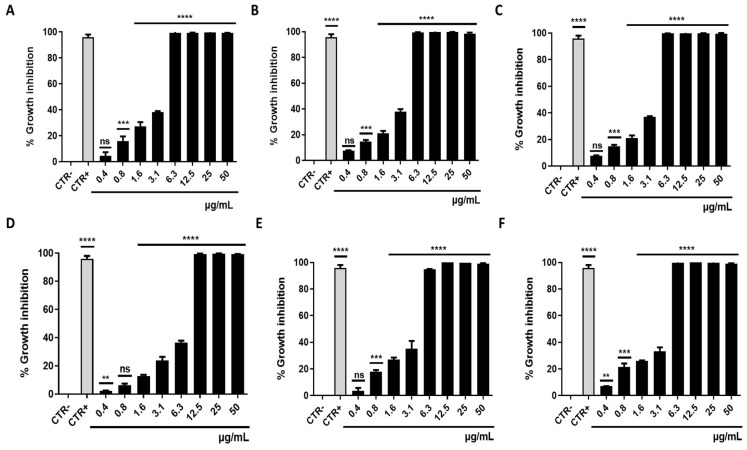
Antimicrobial potential of selamectin. Histogram representations of bacterial inhibition after treatment of (**A**) *S. aureus* ATCC 6538 (****: *p*-value < 0.0001, ***: *p*-value 0.0004, ns: not significant); (**B**) CI1 strain (****: *p*-value < 0.0001, ***: *p*-value 0.0005, ns: no significant); (**C**) CI2 strain (****: *p*-value < 0.0001, ***: *p*-value 0.0002, ns: not significant); (**D**) CI4 strain (****: *p*-value < 0.0001, **: *p*-value 0.0033, ns: not significant); (**E**) CI3 strain (****: *p*-value < 0.0001, ***: *p*-value 0.0002, ns: not significant); and (**F**) CI5 strain (****: *p*-value < 0.0001, ***: *p*-value 0.0004, **: *p*-value 0.0037) with selamectin (50–0.4 µg/mL) for 20h. Vancomycin was used as CTR+, while untreated bacteria were considered the CTR-. *p*-value < 0.05.

**Figure 4:**
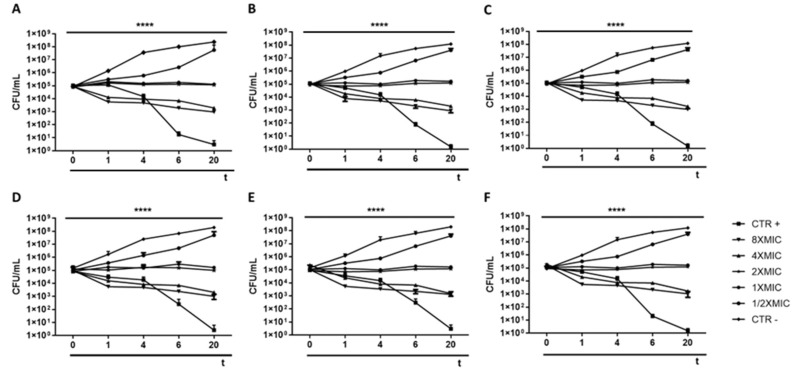
Killing kinetics of selamectin. Curve representations of bacterial load (CFU/mL)after treatment of *S. aureus* ATCC 6538 (**A**), CI1 (**B**), CI2 (**C**), CI4 (**D**) and CI5 (**F**) strains with selamectin at concentrations of 8 × MIC (50 µg/mL), 4 × MIC (25 µg/mL), 2 × MIC (12.5 µg/mL), 1 × MIC (6.3 µg/mL) and 1/2 × MIC (3.1 µg/mL) and of CI3 (**E**) strain with exposure of of 8 × MIC (100 µg/mL), 4 × MIC (50 µg/mL), 2 × MIC (25 µg/mL), 1 × MIC (12.5 µg/mL) and 1/2 × MIC (6.3 µg/mL) selamectin for 0, 1, 4, 6, and 20 h. Vancomycin was used as CTR+, while untreated bacteria were considered the CTR-. ****: *p*-value < 0.0001.

**Figure 5:**
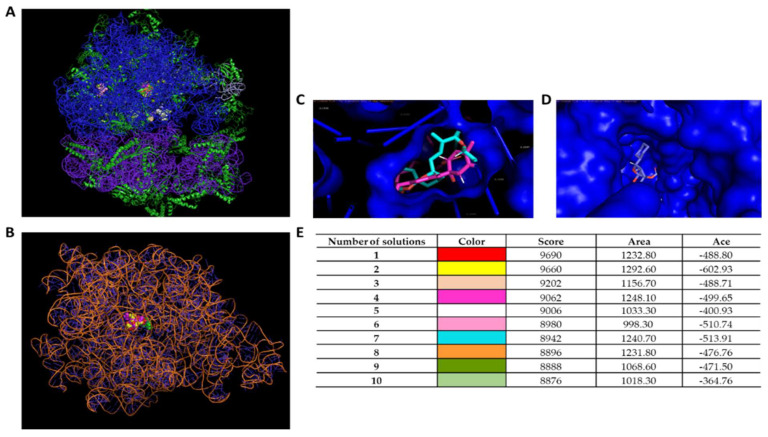
Binding interactions of selamectin with 23S RNA structure derived from a wild-type *S. aureus.* (**A**) Interactions between ribosome and selamectin; (**B**) interactions between 23S rRNA and selamectin; (**C**,**D**) ribosomal channel occupied by selamectin; and (**E**) different interaction models characterized by binding energy scoring.

**Figure 6:**
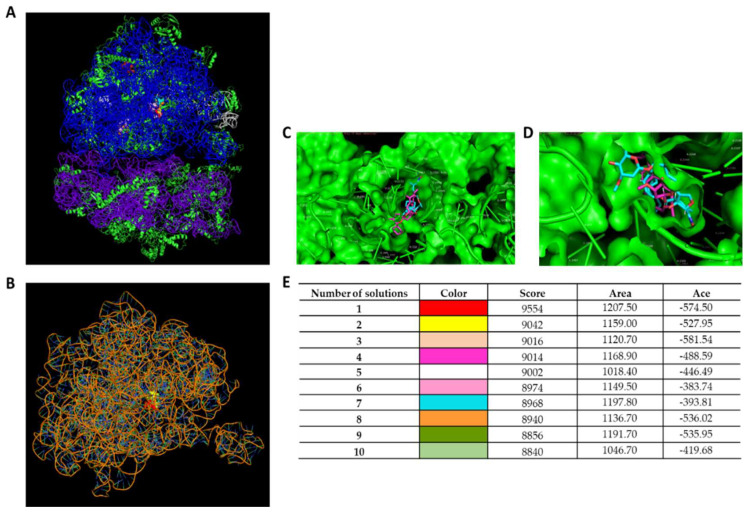
Binding interactions of selamectin with 23S RNA structure derived from a macrolide-resistant *S. aureus*. (**A**) Interactions between ribosome and selamectin; (**B**) interactions between 23S rRNA and selamectin; (**C**,**D**) ribosomal channel occupied by selamectin; and (**E**) different interaction models characterized by binding energy scoring.

**Figure 7:**
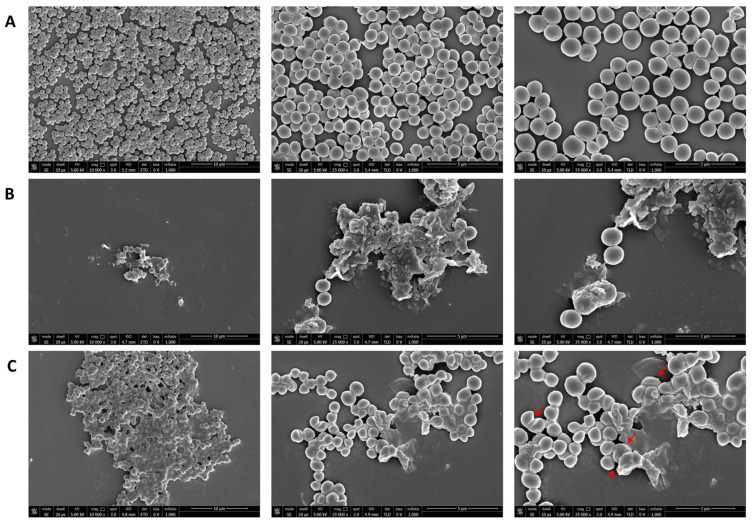
SEM analyses of selamectin impact on bacterial cells. Three different magnifications (10,000, 25,000, and 35,000×) of untreated (**A**) and treated bacterial cells with vancomycin (**B**) and selamectin, at a concentration of 6.3 μg/mL for 20 h (**C**). Red arrows indicate cell damage.

Three different avermectin drugs were screened as potential bacterial growth inhibitors, using the broth microdilution method. The results were reported in Figure 2 and expressed as a percentage of growth inhibition compared to the untreated control. Among the three drugs examined, moxidectin and doramectin did not demonstrate significant antibacterial activity. In contrast, selamectin exhibited a profound impact on the growth of Gram-positive strains at a concentration of 6.3 µg/mL. The inherent resistance of Gram-negative bacteria to selamectin could be attributed to its inability to permeate the bacterial cell. Considering the importance of *S. aureus* in nosocomial infections, the antibacterial action of selamectin was further investigated against clinical isolates (CI1–5) of *S. aureus*. Selamectin displayed potent antibacterial activity against all tested clinical strains, encompassing multisensitive CI1, beta-lactamase producer CI2, quinolones resistant CI4 strain, methicillin-resistant CI5 strain, and CI3 strain with a constitutive resistance phenotype to macrolides, lincosamides, and streptogramin B (cMLSB). Selamectin showed MIC_50_ values of 3.3, 3.4, 3.4, 5.7, 3.5, and 3.4 μg/mL and MIC_90_ of 6.1, 6.0, 6.0, 9.9, 6.6, and 6.4 μg/mL for ATCC 6538, CI1, CI2, CI3, CI4, and CI5, respectively (Figure 3). The kill rate of selamectin was assessed through the time kill test. The bactericidal activity of selamectin was assessed through time-kill tests. The control growth curve (CTR-) displayed an increase in bacterial load over time, indicating no inhibitory effect of the solvent. Conversely, vancomycin treatment for 20 h resulted in a significant reduction of more than 2.8 × 10^4^ compared to the initial bacterial count. Treatment with selamectin at 3.1 µg/mL for ATCC 6538, CI1, CI2, CI4, and CI5, and 6.3 µg/mL for CI3 did not induce relevant growth inhibition compared to CTR-. In contrast, the treatments with 6.3 and 12.5 µg/mL for ATCC 6538, CI1, CI2, CI4, and CI5, and 12.5 and 25 µg/mL for CI3 caused bacterial growth failure, noting no change in the bacteria number compared to the initial bacterial load and indicating the bacteriostatic action of the drug. A decrease in bacterial load occurred after treatment of *S. aureus* strains with the drug at a concentration range of 25–100 µg/mL. Specifically, a 53.8, 45, 52.9, 63.3, and 52-fold decrease for 25 µg/mL exposure, and 110.5, 100, 90, 107.7, and 85.7-fold decrease for 50 µg/mL occurred 20 h after treatment for ATCC 6538, CI1, CI2, CI4, and CI5 strains, respectively. For the CI3 strain, a decrease in bacterial load was observed in response to drug treatment at 50 and 100 µg/mL (Figure 4). A docking simulation of selamectin was performed to identify its probable interactions with 23S RNA structure derived from a wild-type and macrolide-resistant *S. aureus*. Patch Dock provided several interaction model solution options, evaluated through scores and the Atomic Contact Energy (ACE) of the complexes. The findings indicated that selamectin could exhibit a high binding affinity with 23S rRNA molecules. In detail, the drug probably enters the exit tunnel of the nascent peptide of the 50S ribosomal subunit, presumably interfering with the passage of the nascent peptide. Regarding the interactions between the wild-type 23S rRNA structure and selamectin, the most probable solution occupied an area of approximately 1232.80, exhibited a score of 9690, and an ACE of −488.80 kcal/mol (Figure 5). On the other hand, the most likely condition of interaction between selamectin and macrolide-resistant 23S rRNA structure counted a score of 9554 and an ACE of −574.50 kcal/mol, covering an area of 1207.50 (Figure 6). Selamectin-induced bacterial damage was evaluated by SEM (Figure 7). Physical damage with debris formation and reduction in bacterial numbers in response to treatment with 6.3 µg/mL selamectin occurred.

### 3.2. Synergistic Activity of Selamectin with Ampicillin

**Figure 8:**
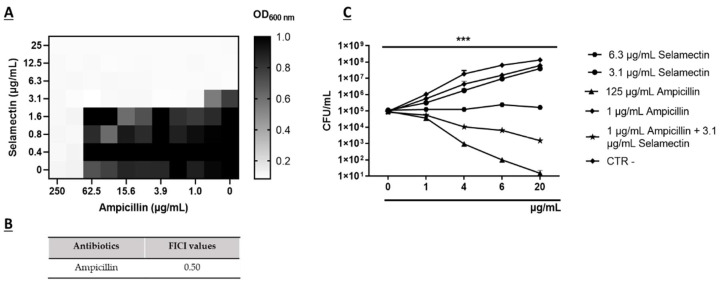
Synergistic activity of selamectin with ampicillin. (**A**) Heat plots of the combination effect of selamectin (25–0.4 μg/mL) with ampicillin (250–0.5 μg/mL) against CI5 strains, stimulated for 20h; CTR+ was untreated cells with drugs; *p*-value < 0.05. (**B**) Table with FICI value calculated for the combination selamectin/ampicillin. (**C**) Killing kinetic curves of bacterial load (CFU/mL) after treatment of CI5 strains with 6.3 and 3.1 μg/mL of selamectin, 125 and 1 μg/mL of ampicillin, and 1 and 3.1 μg/mL of ampicillin and selamectin, respectively. The untreated cell was used as CTR-. ***: *p*-value < 0.0003.

Combinations of two or more antibacterial agents offer a potential avenue for mitigating the emergence of antimicrobial resistance and revitalizing the efficacy of established antibiotics. In this context, we employed a checkerboard assay to explore the potential synergistic activity between selamectin and Ampicillin against the methicillin-resistant *S. aureus* strain (CI5). A FICI value of 0.50 indicated synergy between ampicillin and selamectin. The 3.1 μg/mL dose of the drug reduced the MIC of ampicillin (125 μg/mL) by 125 times against CI5 strains (Figure 8A,B). To verify the nature of the synergistic effect, the killing kinetics of the combination (1 μg/mL ampicillin + 3.1 μg/mL selamectin) and the single components (1 μg/mL and 125 μg/mL ampicillin, 6.3 μg/mL and 3.1 μg/mL selamectin) were evaluated. The findings revealed that 3.1 μg/mL of selamectin and 1 μg/mL of ampicillin induced a growth pattern comparable to CTR-. When 3.1 μg/mL concentration of selamectin was combined with ampicillin at 1 μg/mL, the bacterial survival rate was reduced approximately 63 times compared to the initial bacterial load, after 20 h of treatment (Figure 8C).

### 3.3. Survival of Intracellular S. aureus

**Figure 9:**
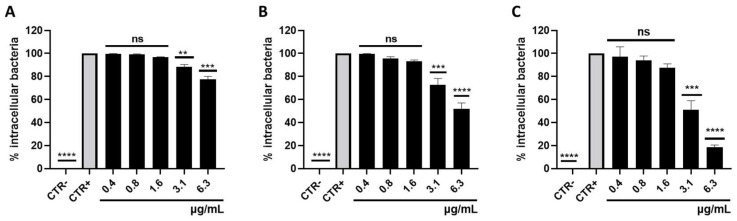
Intracellular survival of *S. aureus* in HaCaT cells. Histogram representations of intracellular bacterial load in HaCat cells after infection with CI5 strains and treatment with selamectin (6.3–0.4 μg/mL) for 0.5 (**A**), 1 (**B**), and 2 (**C**) hours. CTR+ and CTR- were infected and uninfected cells with the bacterial inoculum, respectively. **** *p*-value < 0.0001; ** *p*-value < 0.0040; *** *p*-value < 0.0001; ns: not significant.

*S. aureus* is considered a facultative intracellular bacterium, surviving in the cell cytoplasm for varying periods of time. To evaluate the role of selamectin in counteracting the *S. aureus* intracellular survival in human keratinocytes, gentamicin protection assays were performed in the presence of the drug at concentrations ranging from 6.3 to 0.4 µg/mL. The effect of selamectin against intracellular MRSA followed a dose- and time-dependent trend. After 30 min of exposure, 6.3 and 3.1 µg/mL of the drug reduced the intracellular bacterial load by 22.5 and 11.8%, respectively (Figure 9A). Rates of 52 and 72.7% of bacteria relative to the CTR+ were detected, in response to treatment with selamectin at concentrations of 6.3 and 3.1 μg/mL for 1 h (Figure 9B). Drug exposure at doses of 6.3 and 3.1 µg/mL for 2 h reduced the intracellular bacterial load by 81.3 and 38.5%, respectively (Figure 9C). Non-significant differences compared to CTR+ were found in response to selamectin treatment at concentrations of 0.4, 0.8, and 1.6 μg/mL for different times of exposure, recording intracellular bacterial load rates higher than 87.7%, (Figure 9).

### 3.4. Biofilm Degradation Activity

**Figure 10:**
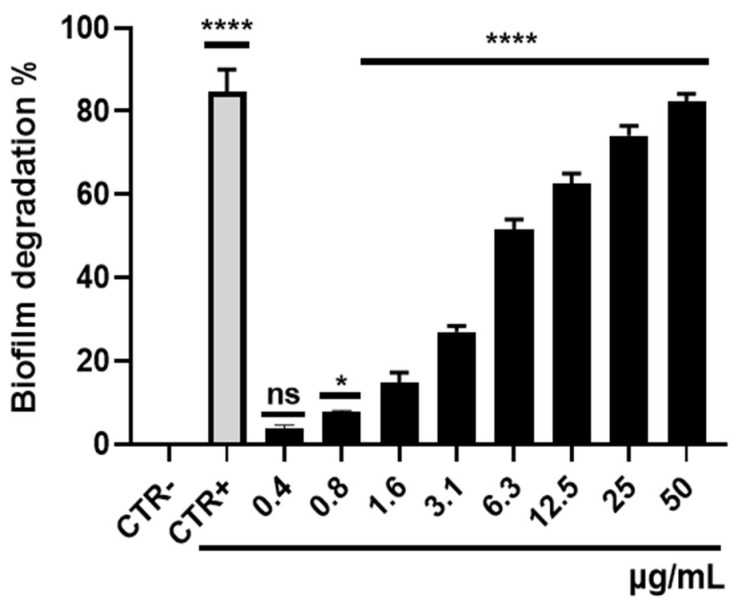
The potential of biofilm biomass disruption by selamectin. Histogram representations of biomass disruption after treatment of biofilm produced by *S. aureus* with selamectin (50–0.4 µg/mL) for 20h; untreated biofilm was CTR-, while vancomycin constituted the CTR+. ****: *p* < 0.0001; *: *p* < 0.005; ns: not significant, relative to drug solvent treated samples (CTR).

**Figure 11:**
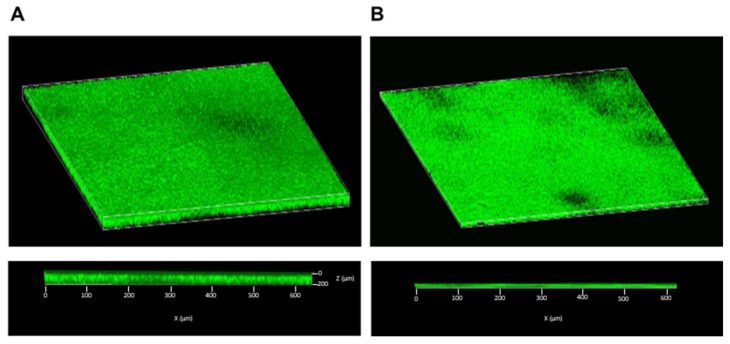
CLSM analysis of selamectin on preformed biofilm of S. aureus. Three-dimensional reconstructions of the Z-stacks (top panel), and 3D cross-section of Z-stacks (bottom panel) of the (**A**) untreated biofilm and (**B**) treated biofilm with selamectin (6.3 µg/mL) for 20 h.

The capacity of *S. aureus* to establish disease-associated biofilms presents a serious challenge in eradicating bacteria from sites of infection. This is attributable to the high tolerance conferred by biofilms to antibiotic interventions, thereby fostering persistent and chronic infections. The impact of selamectin on the integrity of the mature *S. aureus* biofilm was evaluated (Figure 10). Biofilm biomass was quantified by the CV assay after treating the biofilm with 50–0.4 μg/mL of drug for 20 h. The treatment elicited a reduction of up to 82.3% in biomass at the highest concentration tested. At MIC concentrations, the *S. aureus* biofilm lost 51.5% of its mass compared to the drug solvent-treated biofilm. The extrapolated value of selamectin MBEC_50_ was 5.89 μg/mL. CLSM imaging of biofilms, treated with 6.3 µg/mL of selamectin, confirmed this finding. The images documented a thickness of the solvent and drug-treated biofilm equal to 22 and 8 μm, respectively (Figure 11A,B). A reduction in biofilm thickness of 63% and the appearance of lower-density regions occurred in response to treatment. No areas with cell mortality were detected, suggesting that the drug may have a bacteriostatic effect on the preformed *S. aureus* biofilm.

### 3.5. Determination of Cytotoxicity

**Figure 12:**
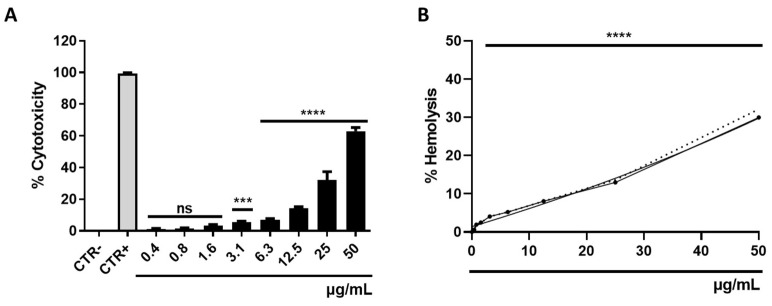
Cytotoxic potential of selamectin. (**A**) Histogram representations of cytotoxicity after treatment of HaCat cell line with selamectin (50–0.4 µg/mL) for 20h; DMSO was used as a positive control (CTR+), while untreated cells were considered the negative control (CTR₋); ****: *p*-value < 0.0001, ***: *p*-value 0.0004, ns: not significant (**A**); and human erythrocytes, the dashed line indicates the DS trend, ****: *p*-value < 0.0001. (**B**) Curve representations of hemolysis after stimulation of erythrocytes with selamectin (50–0.4 µg/mL) for 20h; Triton-X was used as CTR+, while the untreated cell was CTR-; (****: *p*-value < 0.0001). The dotted line indicates the trend of the standard deviation for the tested concentrations.

The assessment of selamectin’s cytotoxic effects was conducted on HaCaT cells and erythrocytes through the utilization of MTT and hemolysis assays, respectively. The range of selamectin concentrations investigated spanned from 50 to 0.4 μg/mL. In both assays, Triton-X (0.1%) and DMSO (100%) were employed as positive controls (CTR+), achieving complete lysis at a rate of approximately 100%. The cytotoxic influence of selamectin on the HaCat cell line increased in a dose-dependent manner, reaching a cell death rate of 63% at the highest concentration tested and a calculated CC_50_ value of 24.94 μg/mL (Figure 12A). Selamectin’s propensity to induce hemolysis was characterized by relatively modest rates, achieving lysis rates of 30% at the concentration of 50 μg/mL. This drug showed no relevant hemolytic activity at concentrations below 12.5 μg/mL (<8.07%) (Figure 12B).

## 4. Discussion

*S. aureus* holds a significant position among clinical pathogens, being responsible for a diverse spectrum of community-based and nosocomial infections. The emergence of multidrug-resistant strains has added complexity to the treatment of *S. aureus* infections, due to the limited therapeutic options available. This challenge has garnered global attention from entities such as WHO and CDC. Of particular concern is the MRSA strain, which is acknowledged as one of the most critical human health pathogens. MRSA’s ability to cause invasive infections and its resistance to multiple antibiotics have necessitated catalytic environmental remediations, monitoring programs, and the pursuit of new drug alternatives [23]. The emergence of resistant bacterial strains is often faster than the development of new antibiotic drugs, therefore, strategies that reduce the time and costs of drug approvals are necessary to provide efficient solutions [24]. Drug repurposing stands as a valuable approach to expedite drug identification, leveraging existing knowledge of toxicity profiles, and facilitating the later stages of clinical evaluation [25]. In recent times, anthelmintic drugs have garnered substantial attention as multi-target agents. Anthelmintics are a series of compounds that exhibit inhibitory activity against helminths [14]. Initially designed for veterinary parasite treatment, these compounds have subsequently been employed in human helminthiasis cases [26]. Some anthelmintics are known to inhibit critical oncogenic pathways, multidrug-resistant bacterial strains, viral infections, and inflammatory activity [27]. Anthelmintic macrolide avermectins have gained significant focus for their multitarget properties. Indeed, Kumar et al. reported that moxidectin and ivermectin exhibit dose-dependent antiviral activity against SARS-CoV2 with EC_90_ values of 7.2 and 5.8 μM, respectively [28]. The anti-inflammatory attributes of ivermectin have been substantiated by multiple studies, which have demonstrated its capacity to inhibit NF-KB transcription, cytokine synthesis, and the modulation of nitric oxide and prostaglandin E2 production [29,30,31]. Although avermectins are macrocyclic lactones, their antibacterial activity remains relatively understudied [32]. Lim et al. documented the effective antibacterial activity of ivermectin, selamectin, moxidectin, and doramectin against 36 strains of *Mycobacterium tuberculosis* with MIC_90_ values below 8 μg/mL [33]. Similarly, Omansen et al. proved the in vitro efficacy of ivermectin and moxidectin on strains of *M. ulcerans*, showing MIC values of 4–8 μg/mL [34]. Moreover, Ashraf et al. established that ivermectin exhibited substantial antibacterial action at concentrations of 6.25 and 12.5 μg/mL against two *S. aureus* isolates [35]. To search for alternative treatments for staphylococcal infections, we screened three avermectins, including selamectin, moxidectin, and doramectin, for their antibacterial potential against *S. aureus*. Our investigation revealed that selamectin displayed that selamectin efficiently inhibited the growth of *S. aureus* ATCC 6538 strains at a concentration of 6.3 µg/mL. Conflicting findings were reported by Lim et al., who did not ascribe any antibacterial efficacy to the aforementioned drug [32]. Due to the efficient antibacterial potential of selamectin, we extended our analysis to clinical strains presenting diverse antibiotic resistance profiles. A mean MIC_90_ value of 6.2 µg/mL was obtained for the multisensitive, beta-lactamase-producing, methicillin- and quinolone-resistant strains. Conversely, the cMLSB-resistant strain exhibited a MIC_90_ value of 9.9 µg/mL. The target sites for macrolides are adenine residues A2058 and A2059, situated within the V region of the 50S ribosomal subunit’s 23S rRNA. Lincosamides and streptogramins B exert their effect through a similar mechanism. The primary mechanism underpinning the MLSB resistance phenotype in *S. aureus* is the modification of the antibiotic binding site by erythromycin ribosome methylase (erm) [36]. The lower efficacy of selamectin against the cMLSB-resistant strain leads us to consider the hypothesis that the drug could interact with 23S rRNA and this interaction could be less effective if the site is methylated. Our in silico analyses showed that this hypothesis deserves to be further investigated. The kinetic data unveiled a bacteriostatic effect of the drug at concentrations of 6.3 and 12.5 µg/mL across all strains, except for CI3, where the drug hindered growth at concentrations of 12.5 and 25 µg/mL. Selamectin exhibited bactericidal activity at concentrations greater than 25 and 50 µg/mL for strains ATCC 6538, CI1, CI2, CI4, CI5, and CI3, respectively. The primary demonstration of selamectin’s antibacterial efficacy has been largely observed against tuberculosis and non-tuberculosis mycobacteria. Specifically, Scherr et al. reported the bactericidal activity of selamectin against *M. ulcerans* with MIC values from 2 to 4 µg/mL [37]. Whereas Muñoz-Muñoz et al. documented the bactericidal action of selamectin against *Mycobacterium marinum*, *Mycobacterium intracellulare,* and *Mycobacterium kansasii* at the same concentrations. In the same study, the drug effectively eliminated strains of *Mycobacterium furtuitum* and *Mycobacterium gordonae* at concentrations between 8 and 16 µg/mL [38]. Moreover, Ezquerra-Aznárez et al. demonstrated its bactericidal potential by identifying it as an inhibitor of decaprenylphosphoryl-β-D-ribose oxidase, a crucial enzyme in the synthesis of mycobacterial arabinogalactan [39]. In contrast to our results, Lim et al. did not report the antibacterial activity of selamectin against *S. aureus* up to 256 µg/mL. The variation in the reported repertoire of antibacterial properties of selamectin can be attributed to the divergence in strains, growth conditions, and methodologies employed [32]. To demonstrate the ability of selamectin to induce cell damage, we performed SEM investigations. This analysis suggested that selamectin induced marked cellular damage, probably due to rearrangements of surface protein structures. The use of multiple antimicrobial agents reduces the occurrence of bacterial resistance and restores the clinical efficacy of some antibiotics on resistant strains [40,41]. Checkerboard assays were conducted to elucidate whether selamectin can exhibit synergistic activity with ampicillin against MRSA strains. The combination of selamectin and ampicillin displayed a synergistic effect, as indicated by a FICI of 0.5, resulting in a substantial reduction of the MRSA bacterial load by approximately 63-fold. In addition to antibiotic resistance, specific bacterial lifestyles, such as invasion of host cells or biofilm growth mode, may also contribute to the persistence or recurrence of *S. aureus* infections [42]. *S. aureus* is recognized as a facultative intracellular pathogen, capable of survival and replication within the host cell cytoplasm. Its intracellular survival ability has been demonstrated in vitro across various cell types, including epithelial and endothelial cells, osteoclasts, keratinocytes, and fibroblasts [43]. Treatment of intracellular *S. aureus* infections represents a serious challenge because most conventional antibiotics, such as beta-lactams and aminoglycosides, remain largely confined to the extracellular space. Selamectin elicited an 81.3% reduction in intracellular bacterial load when applied to infected human keratinocytes at a concentration of 6.3 µg/mL for a duration of 2 h. To our knowledge this is the first report of selamectin activity against intracellular *S. aureus.* Most *S. aureus* isolates form biofilm and this mode of growth entails several advantages for the bacteria engulfed in the biofilm compared to their planktonic counterparts, such as the reduced effectiveness of host defense mechanisms and antibiotics due to poor antibacterial penetration and low bacterial metabolic activity. Concerning the impact of selamectin on mature *S. aureus* biofilms, the CV assay demonstrated a robust reduction in biofilm biomass exceeding 50% in response to treatment at MIC concentration. CLSM data corroborated the antibiofilm activity of selamectin, revealing a significant reduction in the thickness of treated *S. aureus* biofilms, without inducing cell death. It is likely that selamectin acts by influencing the maturation of the biofilm. To date there is no data evaluating the antibiofilm potential of selamectin. Liu et al. evaluated the impact of ivermectin on microbial biofilm reporting no effect on biofilm formation up to 40 μg/mL [44]. An indispensable requirement of a drug is that the effective concentrations are not toxic to the human body. The effect of selamectin was evaluated on human keratinocytes and red blood cells. The drug caused a dose-dependent decrease in cell viability. Selamectin induced hemolysis rates of 5.4% and 8.1%, along with HaCaT cell death rates of 7% and 14.2%, at concentrations of 6.3 μg/mL and 12.5 μg/mL, respectively. The effective concentrations against *S. aureus* remain within non-toxic ranges [38]. The widespread usage of macrocyclic lactones in veterinary medicine has yielded valuable pharmacological insights that could facilitate their adaptation for human applications [45]. Macrolides are widely used for the treatment of large-scale infections. Their utilization is supported by factors such as: (i) proper oral bioavailability; (ii) high intracellular concentration; (iii) range of action limited only to gram-positive strains, this aspect allows the non-alteration of the intestinal microbiota; (iv) drugs generally well tolerated and safe; and (v) strong anti-inflammatory properties that relieves the symptoms due to the release of cytokines at the sites of infection [46,47].

## 5. Conclusions

The application of drug repurposing represents a powerful approach to identifying antibiotics within pre-existing drugs. Our data confirm the efficiency of selamectin in preventing the proliferation of *S. aureus* strains, which includes MRSA, in both the extracellular and intracellular growth stages. The combined effect with ampicillin reveals a significant synergistic result, which translates into a substantial reduction of the bacterial load. Furthermore, shows the ability to perturb the maturation dynamics of S. aureus biofilms. In vitro activity and cytotoxicity studies already conducted in vivo support further evaluation of selamectin for human antibacterial use. The combined efforts of drug repurposing and catalytic environmental remedies hold considerable potential in the fight against antibiotic resistance. These strategies offer avenues for the rapid discovery of new antibacterial agents while simultaneously addressing environmental sources of resistance development. These approaches hold promise in reshaping the healthcare landscape.

## Figures and Tables

**Table 1:** Origins and resistance phenotypes of the bacterial strains used in this study.

Bacterial Species	Strain Number	Resistance Phenotype	Origin
*S. aureus*	ATCC 6538	Multisensitive	ATCC center
*C. striatum*	ATCC BAA-1293	Multisensitive	ATCC center
*S. epidermidis*	ATCC 12228	Multisensitive	ATCC center
*E. coli*	ATCC 11229	Multisensitive	ATCC center
*K. pneumoniae*	ATCC 10031	Multisensitive	ATCC center
*P. aeruginosa*	ATCC 9027	Multisensitive	ATCC center
*S. aureus*	CI1	Multisensitive	Eye
*S. aureus*	CI2	Beta-lactamase producer	Wound
*S. aureus*	CI3	Constitutive resistance to macrolides, lincosamide, streptogramin B	Blood
*S. aureus*	CI4	Quinolones resistance	Sputum
*S. aureus*	CI5	Methicillin resistance	Blood

## Data Availability

The data presented in this study are available on request from the corresponding author.
